# Parental Attitudes and Hesitancy Towards Childhood Influenza Vaccination in Slovakia: A Cross-Sectional Survey of 301 Parents

**DOI:** 10.3390/children13010144

**Published:** 2026-01-20

**Authors:** Peter Kunč, Jaroslav Fábry, Martina Neuschlová, Matúš Dohál, Renata Péčová, Jana Mazuchová, Miloš Jeseňák

**Affiliations:** 1 Jessenius Faculty of Medicine, National Institute of Pediatric Tuberculosis and Respiratory Diseases, Clinic of Pediatric Respiratory Diseases and Tuberculosis, Comenius University in Bratislava, 03601 Martin, Slovakia; jaroslav.fabry@nudtarch.sk; 2 Department of Pathological Physiology, Jessenius Faculty of Medicine, Comenius University in Bratislava, 03601 Martin, Slovakia; martina.neuschlova@uniba.sk (M.N.); renata.pecova@uniba.sk (R.P.); 3 Biomedical Centre Martin, Jessenius Faculty of Medicine, Comenius University in Bratislava, 05981 Bratislava, Slovakia; matus.dohal@uniba.sk; 4 Department of Medical Biology, Jessenius Faculty of Medicine, Comenius University in Bratislava, 03601 Martin, Slovakia; jana.mazuchova@uniba.sk; 5 Department of Paediatrics and Adolescent Medicine, Jessenius Faculty of Medicine, Comenius University in Bratislava, University Hospital in Martin, 03601 Martin, Slovakia; milos.jesenak@uniba.sk; 6 Institute of Clinical Immunology and Medical Genetics, Jessenius Faculty of Medicine, Comenius University in Bratislava, University Hospital Martin, 03601 Martin, Slovakia

**Keywords:** influenza, vaccination, children, parental hesitancy, vaccine hesitancy, Slovakia, public health, pediatric epidemiology, cross-sectional studies

## Abstract

**Highlights:**

**What are the main findings?**

• Critical Vaccination Gap: Despite full health insurance coverage, only 27.6% of Slovak parents expressed willingness to vaccinate their children against influenza, highlighting a profound disconnect between public health policy and parental compliance.
• The Education Paradox: Contrary to global trends, high parental education was not associated with increased vaccine acceptance (*p* > 0.05), suggesting that hesitancy in this region is deeply entrenched across all socioeconomic strata.

**What is the implication of the main finding?**

• Protective Role of Pediatricians: Consulting a pediatrician was identified as the most robust protective factor against hesitancy, increasing the odds of vaccine acceptance six-fold (OR 6.32; 95% CI: 3.54–11.28).
• Dominant Barriers: The primary driver of refusal was fear of adverse effects (70.4%) rather than doubts about efficacy, indicating a need for safety-focused risk communication.

**Abstract:**

**Background/Objectives**: Seasonal influenza imposes a significant burden on pediatric public health. Despite official recommendations and full insurance coverage, vaccination rates among children in Slovakia remain critically low. This study aims to analyze the attitudes, beliefs, and determinants of parental hesitancy regarding childhood influenza vaccination in the post-pandemic context. **Methods**: A single-center cross-sectional survey was conducted between February and March 2025 using convenience sampling among parents of children attending a pediatric immunoallergology center. An anonymous questionnaire collected data on demographics, risk perception, and attitudes. Data from 301 parents were analyzed using descriptive statistics, chi-squared tests, and odds ratios (OR) to identify key predictors of hesitancy. **Results**: Only 27.6% of parents expressed willingness to vaccinate their children, while 42.5% were opposed and 29.9% hesitant. Statistical analysis revealed no significant association between parental university education and vaccination intent (*p* > 0.05), indicating that vaccine hesitancy in this specific setting was present across all educational backgrounds. However, the source of information proved to be a critical determinant: consulting a pediatrician significantly increased the odds of acceptance (OR = 6.32; 95% CI: 3.54–11.28), whereas reliance on the internet and social media was a significant predictor of refusal (OR = 0.29; 95% CI: 0.17–0.50). The primary reported barrier was fear of adverse effects (70.4%), which significantly outweighed doubts about efficacy (30.2%). **Conclusions**: Parental hesitancy in Slovakia is a widespread phenomenon pervasive across all educational backgrounds, driven primarily by safety concerns and digital misinformation. The contrast between the protective influence of pediatricians and the negative impact of digital media underscores that clinical encounters are currently the most effective firewall against hesitancy. Public health strategies must therefore pivot from general education to empowering pediatricians with active, presumptive communication strategies.

## 1. Introduction

Seasonal influenza is a contagious viral illness that poses a considerable threat to public health worldwide. While often trivialized and mistaken for a common cold, influenza can lead to severe complications, hospitalization, and even death, particularly in vulnerable populations [1]. Children, especially those under five years of age, are not only at a higher risk of developing severe disease with systemic symptoms but also act as major drivers of influenza transmission within communities [2]. Due to factors such as less developed respiratory hygiene, prolonged viral shedding (which can last more than 10 days), and close contact in school environments, children effectively spread the virus. This poses a significant risk not only to their peers but also to other vulnerable individuals in their surroundings, such as infants too young to be vaccinated and the elderly, for whom influenza can be fatal [3]. Complications in children are frequent and include otitis media, pneumonia, and severe neurological conditions such as febrile seizures and encephalitis [1].

Vaccination remains the most effective strategy for preventing influenza and its associated complications [4]. In Slovakia, health authorities, including the Public Health Authority of the Slovak Republic (ÚVZ SR), strongly recommend annual influenza vaccination for all children from 6 months of age. Several types of modern, trivalent vaccines are available. Standard inactivated vaccines (IIV), administered via injection, are fully covered by public health insurance for all children. Additionally, a live attenuated intranasal vaccine (LAIV), administered as a nasal spray, which is often preferred for children due to its needle-free application, is also available and is fully or partially reimbursed depending on the child’s age, further removing financial barriers [5].

Despite these comprehensive measures, vaccination coverage in Slovakia is among the lowest in Europe [6]. The overall vaccination rate for the entire population for the 2024/2025 season remains critically low, at just 5.2% [7]. However, national data from recent seasons indicate a positive, albeit modest, trend, with the number of vaccinated children in the 0–15 age group increasing from 14,792 (1.6% coverage) in the 2023/2024 season to 23,306 (2.5% coverage) in the 2024/2025 season [7]. Nevertheless, according to the European Centre for Disease Prevention and Control (ECDC) data for the 2023-24 season, the overall vaccination rate for children in Slovakia was approximately 2% [5]. This figure is dwarfed by rates in other European countries such as Finland (38.9%), Spain (36.1%), and Ireland (16%), highlighting a profound public health challenge. This low uptake falls dramatically short of the 75% target for at-risk groups set by the World Health Organization (WHO) and the European Union (Council of the European Union, 2009) [8].

This critical gap between official recommendations and public behavior is largely fueled by parental vaccine hesitancy, a complex phenomenon influenced by a multitude of factors including perceptions of risk, trust in healthcare systems, and the overwhelming spread of misinformation [9,10]. The experience of the COVID-19 pandemic has further complicated this landscape, fundamentally altering public perceptions and introducing new barriers to routine immunizations [11]. Understanding the specific drivers of this hesitancy within the Slovak context is essential for developing effective public health interventions.

This study aims to investigate the attitudes, knowledge, and beliefs of parents in Slovakia regarding childhood influenza vaccination. By analyzing data from a cohort of 301 parents, we seek to identify the primary reasons for vaccine acceptance, refusal, and hesitancy, with a particular focus on the role of pediatricians and information sources. Understanding these barriers is crucial for epidemiological modeling and predicting future outbreak patterns in Central Europe.

While vaccine hesitancy is well-documented in Western Europe and North America, there is a scarcity of post-pandemic data from Central Europe, specifically Slovakia. This region is characterized by unique socio-political dynamics and historically lower uptake of voluntary vaccines compared to mandatory ones. Addressing this gap is critical, as findings from Western democracies may not be directly translatable to post-communist settings with different healthcare-seeking behaviors. Therefore, this study aims to analyze the attitudes, beliefs, and determinants of parental hesitancy regarding childhood influenza vaccination in this specific context.

## 2. Materials and Methods

### 2.1. Study Design and Participants

This questionnaire-based cross-sectional study was designed to determine parental attitudes and hesitancy towards childhood influenza vaccination. The target population consisted of parents or legal guardians of children visiting the pediatric outpatient immunoallergology unit in the National Institute of Pediatric Tuberculosis and Respiratory Diseases, Dolny Smokovec, for routine check-ups or minor illnesses between February and March 2025. Exclusion criteria were defined as (1) parental refusal to participate, (2) submission of an incomplete questionnaire, or (3) absence of the child’s legal guardian. A total of 301 parents voluntarily and anonymously participated in the study by convenience sampling.

The sample size (n = 301) was determined by convenience sampling, reflecting the flow of eligible parents visiting the center during the seasonal study period. We acknowledge that recruitment from a specialized pediatric center introduces selection bias, potentially overrepresenting mothers and parents of children with chronic respiratory conditions compared to the general population.

### 2.2. Questionnaire

The questionnaire was specifically designed to reflect the socio-cultural context of Slovakia while incorporating elements from validated tools, such as the Parent Attitudes about Childhood Vaccines (PACV) survey [12]. The instrument underwent content validation by a panel of pediatric experts (co-authors) and was pilot tested on a sample of 10 respondents to ensure clarity and relevance prior to distribution. The first section gathered sociodemographic characteristics of both parents and their children. Subsequent sections delved into parental attitudes, covering domains such as:

• Health status and experience with chronic diseases.
• Perception of influenza severity in children.
• Self-assessed level of information and primary sources of information regarding vaccination.
• Attitude towards vaccinating their child/children against influenza.
• Specific reasons for vaccination (for accepting parents) or against it (for refusing parents), and factors influencing hesitant parents.
• Opinions on the safety and efficacy of the influenza vaccine and the role of the pediatrician.

The complete questionnaire in its original Slovak version, as administered to respondents, along with the translated English version, are available as Supplementary Materials. To ensure internal validity, the reliability of the attitude-related items (trust, efficacy, safety perception) was assessed using Cronbach’s alpha analysis, which demonstrated good internal consistency (α = 0.81).

### 2.3. Data Collection and Statistical Analysis

Data were collected directly from participants. Upon providing informed consent, participants completed the questionnaire electronically using a handheld tablet interface via the Google Forms^®^ platform (Google LLC, Mountain View, CA, USA), ensuring direct and anonymous data entry. Initially, 320 questionnaires were collected; however, following a rigorous data validation process, 19 incomplete submissions were excluded, resulting in a final analytical sample of 301 questionnaires. This method ensured direct engagement with the target population. Participation was voluntary and anonymous and was conducted without the presence of medical personnel to ensure that responses were not influenced by their presence and to encourage candid answers. The collected data from the 301 completed questionnaires were analyzed using descriptive statistics. Data were analyzed using Jamovi, a statistical software package (The Jamovi Project, 2023, Sydney, Australia, Jamovi Version 2.3.28.0) built on the R statistical language. Frequencies and percentages were calculated for each response, and the results were interpreted to identify key trends, barriers, and facilitators related to the vaccination decision-making process. To determine the strength of associations between categorical variables (e.g., parental education level and vaccination intent), Pearson’s chi-squared test (χ^2^) was performed. Odds ratios (OR) with 95% confidence intervals (CI) were calculated to quantify the magnitude of these associations. To visualize hypothesized causal pathways and distinguish between confounders (e.g., education) and mediators (e.g., trust, fear), a Directed Acyclic Graph (DAG) was constructed using the DAGitty software (v3.1, Johannes Textor, Radboud University, Nijmegen, The Netherlands) logic to distinguish between confounders and mediators. The model posits that the influence of information sources on vaccination intent is mediated through trust and safety concerns, while past vaccination history acts as a strong direct predictor. All statistical tests were two-tailed, and a *p*-value of <0.05 was considered statistically significant.

### 2.4. Ethical Considerations

The study was conducted in accordance with the Declaration of Helsinki. The study was approved by the Ethics Committee of the National Institute of Pediatric Tuberculosis and Respiratory Diseases in Dolny Smokovec, Slovakia (ID 2025046). Anonymity and confidentiality of the participants were guaranteed.

## 3. Results

### 3.1. Participant and Child Demographics

**Figure 1:**
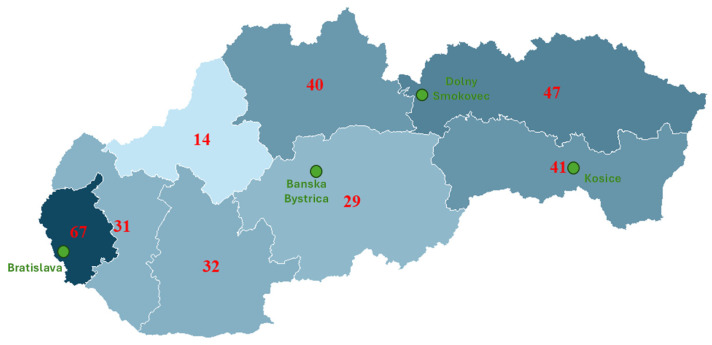
Geographical Distribution of Survey Respondents by Region. The map highlights the study location (Dolny Smokovec) and major urban centers (Bratislava, Banska Bystrica, Kosice) for orientation. (Map of Slovakia showing proportional representation of respondents from different regions).

A total of 301 parents participated in the survey. The majority of respondents were women (89.0%), predominantly in the 35–44 (47.5%) and 25–34 (46.2%) age groups. The sample was characterized by a high level of education, with nearly two-thirds (64.8%) holding a university degree. The geographical distribution of the respondents by region is shown in Figure 1.

The 301 respondents were parents to a total of 443 children. The most represented age group among the children was 5–9 years (34.3%). A significant portion of families had more than one child, and a substantial number of children (24.3%) had a chronic health condition, placing them in a high-risk group for severe influenza. Detailed sociodemographic characteristics are presented in Table 1.

### 3.2. Parental Perception and Information Level

Most parents (over 70%) perceived influenza as a potentially serious disease in children. However, more than a quarter of respondents still considered it a common, non-threatening illness. When asked to identify groups most at risk of influenza complications, parents correctly recognized seniors (82.7%) and the chronically ill (79.4%) as the primary risk categories. In contrast, only 57.1% of parents identified small children as a high-risk group, and even fewer (40.9%) recognized pregnant women as being particularly vulnerable. This highlights a significant gap in risk perception, where parents underestimate the specific threat influenza poses to the pediatric population.

Regarding information, a significant knowledge gap was identified. While a majority of parents felt informed, over 40% stated they had only partial or no information about the possibility of vaccinating their children against influenza. The pediatrician was the most frequently cited source of information, with 79.4% of parents identifying them as such. However, the internet was a close second, used by 54.5% of respondents, highlighting a dual-channel information environment. Other significant sources included family and friends (25.2%) and traditional mass media (23.9%), while social media was cited by 15.0% of parents. Notably, when asked specifically about the existence of a painless, intranasal spray vaccine (LAIV), a significant information deficit emerged: while a majority (56.1%) were aware of this option, almost four in ten parents (37.9%) had no information about it, and 6.0% could not recall. This information deficit is further underscored by the finding that a striking 65.1% of parents were unaware that influenza vaccination is recommended for children as young as 6 months, a key high-risk group.

### 3.3. Vaccination Decision and Influencing Factors

**Figure 2:**
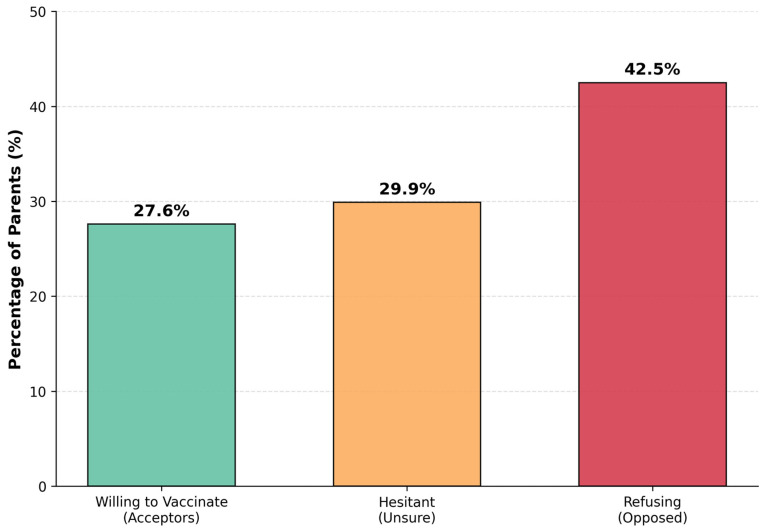
Parental willingness to vaccinate children against influenza (n = 301). The bar chart illustrates the distribution of parents into three categories: Acceptors (27.6%), Hesitant (29.9%), and Refusers (42.5%).

The major finding of the survey was a low parental acceptance of the influenza vaccine. The respondents were divided into three distinct groups: 27.6% (n = 83) were willing to vaccinate their child, while a larger group of 42.5% (n = 128) were opposed, and a significant portion, 29.9% (n = 90), remained undecided or hesitant (Figure 2). We further stratified the data to analyze the impact of parental education on vaccine acceptance. Univariate analysis revealed no statistically significant association between university education and a positive attitude towards vaccination (**χ^2^** = 1.14, *p* = 0.285). Specifically, the proportion of vaccine-hesitant or refusing parents was comparably high among university graduates (71.3%) and those with secondary education (74.4%). The calculated Odds Ratio (OR = 0.85; 95% CI: 0.51–1.42) indicates that holding a university degree did not serve as a significant protective factor against vaccine hesitancy in our cohort. This finding suggests that barriers to vaccination in Slovakia are deeply entrenched and cut across socioeconomic lines. A detailed breakdown of the differing attitudes, beliefs, and primary motivations within these three groups is presented in Table 2.

Analysis of the entire cohort reveals a clear hierarchy of factors influencing parental decisions (Table 3). A recommendation from a doctor stands out as the most powerful motivator for vaccination (58.8%), while the fear of adverse effects is the most significant barrier (70.4%). When considering the perceived benefits of vaccination, parents prioritized the mitigation of the disease’s course (72.1%) over complete prevention of the illness (56.1%). In terms of information seeking behavior, our analysis identified a stark dichotomy. Reliance on a pediatrician as a primary information source emerged as a robust protective factor against vaccine hesitancy. Parents who consulted their pediatrician had significantly higher odds of accepting the vaccine compared to those who did not (OR = 6.32; 95% CI: 3.54–11.28; *p* < 0.001). Conversely, reliance on the internet and social media was significantly associated with vaccine refusal (OR = 0.29; 95% CI: 0.17–0.50; *p* < 0.001). Furthermore, the strongest predictor of future vaccination intent was the child’s vaccination history; parents whose children had been vaccinated against influenza in previous seasons were significantly more likely to intend to vaccinate again (OR = 68.73; 95% CI: 20.39–231.62; *p* < 0.001), highlighting the importance of establishing early adherence habits.

### 3.4. Multivariable Analysis of Determinants

To control for confounding factors, we performed a binary logistic regression analysis (Table 4). The model evaluated the association between parental willingness to vaccinate (dependent variable) and key demographic and behavioral predictors. After adjustment, university education (aOR = 0.96; *p* = 0.910) and the presence of a chronic disease in the child (aOR = 1.05; *p* = 0.898) were not significantly associated with vaccination intent. However, the source of information remained a critical independent predictor. Consulting a pediatrician significantly increased the odds of acceptance (aOR = 6.67; 95% CI: 3.02–14.74), whereas reliance on the internet and social media was a significant predictor of refusal (aOR = 0.30; 95% CI: 0.15–0.58). A history of previous influenza vaccination was the primary predictor of future intent (aOR = 18.84; *p* < 0.001). This high odds ratio reflects a high degree of behavioral consistency, indicating that parents who have previously overcome barriers to vaccination have established a routine behavior.

To further distinguish between ‘hesitant’ parents (those answering ‘I don’t know’) and ‘refusing’ parents, a multinomial logistic regression was performed (Table 5). The analysis revealed that consulting a pediatrician significantly increased the likelihood of a parent being ‘Hesitant’ rather than ‘Refusing’ (Relative Risk Ratio = 2.45; *p* = 0.015). This suggests that physician engagement may prevent parents from sliding into hard refusal, keeping them in a window of opportunity for intervention.

### 3.5. Trust in Vaccine Safety and Efficacy

Parental trust in the influenza vaccine is critically low. A staggering 44.5% of parents have concerns about its safety or believe it is not completely safe, with only 22.6% considering it safe. The remaining 32.9% were unable to assess its safety or lacked information.

**Figure 3:**
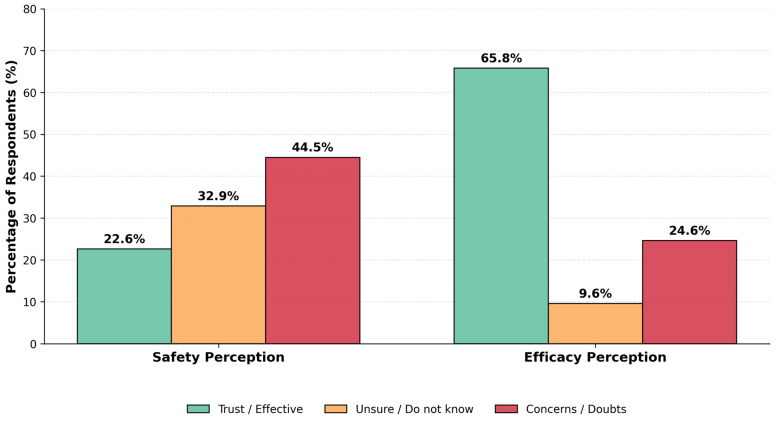
Parental Trust in the Safety and Efficacy of Influenza Vaccines for Children (n = 301). The grouped bar chart compares parental attitudes regarding vaccine safety (left cluster) and efficacy (right cluster). Responses are stratified into three categories: Trust/Perceived as Effective (green bars), Uncertainty/Do not know (orange bars), and Concerns/Doubts (red bars). Values represent the percentage of respondents in each category.

In contrast to safety concerns, parental confidence in vaccine efficacy was notably higher. As shown in Figure 3, the majority of respondents (65.8%) perceived the vaccine as effective (combining those who considered it ‘sufficiently’ or ‘partially’ effective). Explicit doubts regarding efficacy were reported by 24.6% of parents, while the remaining 9.6% were unable to assess it.

### 3.6. The Role of the Pediatrician and Experience with Adverse Reactions

The survey unequivocally highlighted the pivotal role of the pediatrician in the parental decision-making process. An overwhelming majority of parents (86.3%) confirmed that a recommendation from their pediatrician would influence their decision (49.8% “very much” and 36.5% “partially”). Furthermore, 69.1% of parents explicitly stated they are interested in receiving more information directly from their pediatrician, confirming a high demand for proactive communication from trusted healthcare professionals.

To contextualize parental fears, the survey also inquired about direct experiences with adverse reactions following influenza vaccination. An overwhelming majority of parents (97%) reported that their child had never experienced an adverse reaction. Of the small fraction (3%, n = 9) who did report a reaction, the most common events were mild and transient, such as fever or increased temperature, which were reported by 6 of the 9 respondents. Notably, one parent cited a perceived link between the vaccine and a neurodevelopmental disorder (ADHD/autism), illustrating the presence of serious, albeit scientifically unfounded, concerns within the parent community.

## 4. Discussion

This study provides critical insight into the multifaceted nature of parental hesitancy towards childhood influenza vaccination in Slovakia, a country with one of the lowest uptake rates in Europe. Our findings reveal that the decision not to vaccinate is not born from a single issue but from a confluence of low-risk perception, profound mistrust in the vaccine’s safety and efficacy, and a significant information deficit, all of which are amplified by a complex socio-cultural environment [13,14]. Our findings of critically low uptake align with the broader phenomenon of vaccine backsliding currently observed across the WHO European Region. Recent ECDC data indicate a concerning decline in coverage even for routine pediatric vaccines (such as MMR and DTP) in the post-COVID-19 era. However, a crucial distinction must be made: unlike mandatory vaccines, where coverage remains relatively high due to legislative enforcement, voluntary influenza vaccination suffers disproportionately from this general erosion of trust. In this context, the hesitation we observed is not merely an influenza-specific issue but a symptom of a wider crisis in public confidence [15].

**Figure 4:**
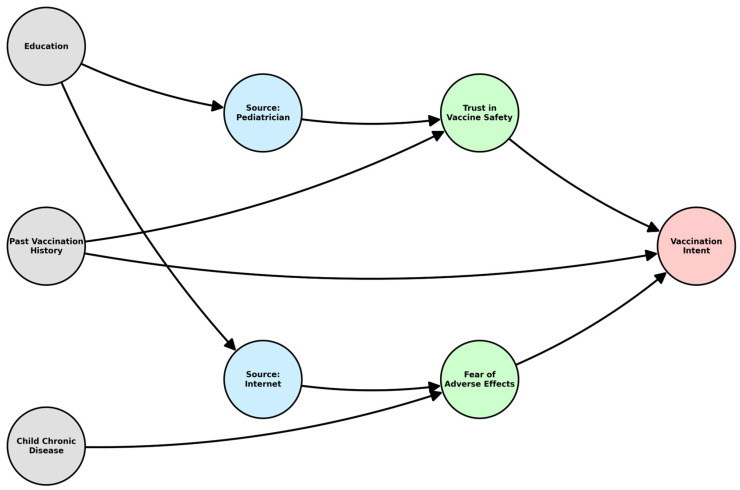
Directed Acyclic Graph (DAG) illustrating the determinants of vaccination intent. he model visualizes the protective pathway from pediatricians to trust versus the negative pathway from internet sources to fear, with past vaccination history acting as a strong direct predictor.

The core drivers of hesitancy identified in our Slovak cohort align remarkably well with the international body of evidence. The triad of low disease risk perception, concerns over vaccine safety, and doubts about its efficacy forms the cornerstone of parental opposition globally [9,16]. Our finding that over a quarter of parents perceive influenza as a “common illness” mirrors studies like Smith et al., which identified low perceived necessity as a key psychological barrier [17]. This corresponds with health behavior frameworks like the Health Belief Model, where perceived severity and perceived benefits are crucial determinants of action; our findings are consistent with recent post-pandemic research showing these factors are central to parental decisions [18]. This underestimation of risk is further compounded by our finding that while parents generally acknowledge the severity of influenza, a substantial portion (42.9%) do not perceive their own children as a primary high-risk group for complications, placing them behind seniors and the chronically ill in their risk assessment. This perceptual gap is a critical barrier: if parents do not see their child as being particularly vulnerable, the motivation for a specific preventive measure like vaccination is fundamentally weakened. This is particularly concerning given that nearly a quarter of the children in our cohort had chronic conditions, placing them at high risk. The hypothesized causal pathways are visualized in the Directed Acyclic Graph (Figure 4).

The overwhelming fear of adverse effects and doubts about efficacy are the most cited reasons for hesitancy worldwide [19]. Our study confirms this pattern in the Slovak context, where the fear of adverse effects was the most significant concern, cited by over 70% of all parents, more than double the number who cited doubts about efficacy (30.2%). This high level of fear stands in contrast to the actual reported experience within our cohort, where 97% of parents stated their child had never had an adverse reaction to the influenza vaccine. This disconnect between perceived risk (70.4% are concerned) and lived experience (3% reported a reaction) is a powerful illustration of how vaccine hesitancy is often driven by abstract fears and misinformation rather than direct negative outcomes. Furthermore, the nature of the few reported reactions, mostly mild fever, is consistent with the known and expected immunogenic response to the vaccine. The isolated report linking vaccination to autism, while representing a single respondent, is critically important as it provides a concrete example of the persistence of debunked yet powerful myths that fuel the most profound parental anxieties. Crucially, a large US national survey by Kempe et al. demonstrated that while safety concerns for influenza and routine childhood vaccines were nearly identical, hesitancy towards influenza vaccination was over four times higher (25.8% vs. 6.1%) [20]. The authors concluded this was largely driven by a profound lack of confidence in the influenza vaccine’s effectiveness, a finding that strongly resonates with our results, where only 13.6% of parents believed the vaccine to be sufficiently effective. These concerns are often amplified by misconceptions, such as the fear that the vaccine can cause influenza or that it “overloads” the immune system, notions that are prevalent in anti-vaccination discourse [21].

A crucial finding of our study is the profound paradox surrounding the role of the pediatrician. On one hand, parents in our cohort identified the pediatrician as the most important source of information, with an overwhelming 86% stating that a doctor’s recommendation would influence their decision. This aligns perfectly with our finding that a doctor’s recommendation is the single most powerful motivating factor for vaccination, cited by nearly 60% of parents. Furthermore, this is highly consistent with international literature, which repeatedly confirms that a clear, strong recommendation from a trusted healthcare provider is one of the most powerful predictors of vaccine acceptance [22,23]. On the other hand, the actual vaccination rate remains abysmal. This discrepancy suggests that the potential of the patient-pediatrician relationship is not being fully leveraged in Slovakia. This is where insights from a comprehensive Cochrane review on improving adolescent vaccination uptake become particularly relevant [24]. The review found high-certainty evidence that health education improves vaccine uptake. Our finding that a majority of parents desire more information from their pediatrician suggests that a structured educational intervention, delivered by a trusted professional, could be a highly effective strategy in the Slovak context. International research emphasizes that it is not just any recommendation, but a clear, consistent, and proactive one that makes a difference [19]. The lack of such a strong “cue to action,” a factor also highlighted as critical by Cao et al., likely explains why high theoretical trust in pediatricians does not translate to high vaccination uptake in the Slovak context [18]. The pivotal role of the pediatrician is further quantified by our statistical findings. The six-fold increase in the odds of vaccine acceptance (OR 6.32) among parents utilizing pediatricians as an information source provides empirical evidence that the physician’s influence is not merely theoretical but translates into tangible behavioral intent. This contrasts sharply with the negative impact of digital information sources (OR 0.29), confirming that the ‘digital echo chambers’ described in the literature [25,26] actively erode vaccine confidence in the Slovak population. This dichotomy suggests that clinical encounters are currently the most effective, albeit underutilized, firewall against the spread of misinformation. The communication may be happening too passively; a simple offer of the vaccine, without a strong, proactive recommendation, can be interpreted by a hesitant parent as a lack of urgency or conviction on the part of the physician. This passivity fails to counteract the powerful emotional narratives of risk that parents encounter elsewhere. This leads to the critical issue of the information ecosystem. Our survey confirms that while the pediatrician remains the most trusted and most frequently cited source of information (79.4%), the internet is a powerful competitor, used by a majority of parents (54.5%) to seek health information. This creates a complex dynamic where professional medical advice is often weighed against, or filtered through, information of variable quality found online. The significant reliance on family and friends (25.2%) and traditional mass media (23.9%) further complicates this landscape, creating echo chambers where misinformation can thrive [13]. A recent study among highly educated Jordanian parents by Al-Iede et al. further illuminates this dynamic, finding that while healthcare workers were the most common source of information for non-hesitant parents, hesitant parents were significantly more likely to rely on social media and friends [27]. This underscores the challenge of penetrating closed social circles with evidence-based information. However, the role of social media may be more nuanced. A recent study also found that while general reliance on social media can foster hesitancy, exposure to specific information, such as testimonials about severe cases or news of hospital strain, can decrease parental hesitancy [18]. This suggests that the content and framing of information are critical. The same study also uncovered a potential gender difference, where fathers were more influenced by statistical data on rising case numbers, while mothers were not, indicating that effective communication strategies may need to be tailored. A pivotal finding of our study is the statistical absence of a correlation between parental education and vaccine uptake (*p* > 0.05). This challenges the traditional paradigm that vaccine hesitancy is primarily a consequence of lower health literacy. Instead, our results align with the ‘highly educated yet hesitant’ phenomenon described in the recent literature [28,29]. In post-communist Central European contexts, higher education may paradoxically correlate with increased skepticism towards institutional recommendations and a greater confidence in one’s ability to research ‘alternative’ health information. This implies that public health strategies relying solely on fact-based education may fail to reach this demographic, necessitating more value-based communication strategies. The lack of association between education and acceptance may also reflect specific cultural dynamics in post-communist Central Europe. In these settings, a historical skepticism toward state-mandated guidelines often persists. Highly educated parents may feel more confident in conducting their own ‘research’ on alternative platforms, paradoxically making them more susceptible to sophisticated misinformation than lower-education groups who may strictly follow physician advice [30]. This aligns with findings that higher functional health literacy, if coupled with low trust in official sources, can lead to more critical questioning and a greater ability to seek out and consume sophisticated misinformation, thereby reinforcing hesitancy [31].

A particularly insightful finding from our survey is the hierarchy of perceived benefits of vaccination. That the majority of parents (72.1%) view “mitigating the course of influenza” as the primary benefit, ahead of complete prevention (56.1%), suggests a sophisticated, and perhaps realistic, understanding of the vaccine’s function. This contrasts with the binary “it works, or it doesn’t” thinking often assumed in public health messaging. It indicates that parents may be receptive to communication that honestly frames the vaccine not as an infallible shield but as a crucial tool for reducing severity and preventing the most dangerous outcomes. This nuanced perspective could be a powerful entry point for conversations with hesitant parents, shifting the focus from the impossibility of 100% prevention to the tangible benefit of avoiding hospitalization and severe complications.

The impact of the COVID-19 pandemic on vaccination attitudes is a critical contextual factor. This polarization is consistent with the phenomenon of ‘pandemic fatigue’ [32,33], where prolonged exposure to public health mandates and the saturation of health-related information have led to desensitization and resistance in a subset of the population. Conversely, for others, the pandemic has served as a ‘teachable moment,’ reinforcing the tangible risks of respiratory viral infections. Our survey reveals a complex and polarized effect on Slovak parents. While the majority (68.4%) reported that the pandemic did not change their stance on influenza vaccination, a significant minority experienced a shift in their views. Notably, this shift was almost evenly split, with 16.6% of parents reporting a more positive attitude towards vaccination, likely due to an increased awareness of the severity of respiratory viruses, while 15.0% reported a negative shift, potentially fueled by concerns over expedited vaccine development processes, widespread misinformation, and a general erosion of trust in public health institutions during the pandemic [34]. This polarization suggests that the pandemic did not create a uniform trend but rather intensified pre-existing beliefs, pushing some parents towards greater acceptance and others towards deeper hesitancy.

The Slovak context appears to be characterized by a particularly deep-seated trust deficit. The preference for “natural immunity,” expressed by a quarter of refusing parents in our survey, is not merely a scientific misunderstanding but often part of a broader ideological stance that favors “natural” approaches and distrusts medical or pharmaceutical interventions [13]. This preference may also influence the choice between vaccine types. A longitudinal study by Yuan et al. found that parents with higher vaccine hesitancy showed a significantly greater preference for the non-invasive nasal spray LAIV over the injectable IIV. The authors link this to the perception that LAIV is less invasive and aligns better with values of naturalness and concerns about overmedication [35]. This makes our finding that almost 40% of Slovak parents are unaware of the existence of this painless, child-friendly option a critical public health failure. The lack of awareness about a vaccine that directly addresses some of the core ideological and practical barriers (such as fear of needles) represents a significant missed opportunity to engage hesitant parents and improve vaccination uptake. This is compounded by the fact that nearly two-thirds of parents (65.1%) did not know that vaccination is recommended for infants from 6 months of age, leaving the most vulnerable age group unprotected due to a simple lack of basic information. The mentioned sentiment, combined with a general skepticism towards state institutions, creates fertile ground for vaccine refusal. When public health is politicized, as it has been in recent years, this trust erodes even further, making any official recommendation seem suspect. Furthermore, our findings are consistent with strong evidence showing that parents’ own vaccination status is a major predictor of their child’s vaccination [36]. Moreover, a complex study by Cao et al., among others, found a particularly strong association, with parents hesitant for themselves being over four times more likely to be hesitant for their children [18]. This is reinforced by the findings of Al-Iede et al., where parental compliance with national vaccination guidelines was one of the strongest positive independent predictors of lower vaccine hesitancy [27]. This highlights a self-perpetuating cycle of low vaccination coverage that passes from one generation to the next. The Cochrane review also found that multi-component interventions targeting both parents and providers may improve vaccine uptake, suggesting that a coordinated approach is likely more effective than isolated efforts [24]. Crucially, our results underscore the need for a paradigm shift in clinical practice. The gap between high parental trust in pediatricians and low actual uptake suggests that the current passive communication model is insufficient.

We recommend that clinicians shift from a ‘participatory’ approach (asking parents what they want to do) to a ‘presumptive’ initiation of vaccination discussions (presenting the vaccine as a routine standard of care) [37,38]. The effectiveness of such presumptive communication is supported by intervention studies in similar settings. For instance, Opel et al. demonstrated that a presumptive initiation format significantly increased parental acceptance compared to a participatory approach. Adapting these evidence-based training models for Slovak pediatricians could provide the necessary tools to convert theoretical trust into practical uptake [39]. Evidence suggests that a presumptive recommendation significantly increases vaccine acceptance. Pediatricians should not wait for parental inquiries but must actively normalize influenza vaccination as an integral component of preventive pediatric care, thereby bridging the gap between trust and action.

The limitations of this study must be acknowledged. The primary limitation is the use of a non-random, convenience sample recruited from a single, specialized pediatric immunoallergology outpatient unit. It is also necessary to address the disparity between the reported willingness to vaccinate in our survey (27.6%) and the critically low national coverage (approx. 2.5%). We attribute this discrepancy to three concurrent factors. First, selection bias: parents visiting a specialized center for respiratory diseases are likely more sensitized to health risks than the general population. Second, social desirability bias may lead respondents to overestimate their intent in a survey setting to align with perceived medical norms. Third, the well-documented ‘intention-behavior gap’ suggests that a positive theoretical attitude often fails to translate into practical action due to logistical barriers or procrastination. This introduces a significant selection bias, and therefore the findings cannot be generalized to the entire Slovak population. Our cohort consisted of parents of children often managing chronic conditions and was characterized by an exceptionally high level of university education (64.8%), which is not nationally representative. This specific demographic may hold different views compared to the general population; for instance, direct experience with chronic illness could increase vaccine acceptance, while concerns about a “sensitive” immune system could heighten hesitancy. Nevertheless, the high degree of hesitancy observed even within this highly educated group is a significant finding, challenging the assumption that higher education directly correlates with vaccine acceptance. Future research using a randomized, nationally representative sample is needed to validate these findings and accurately map the determinants of vaccine hesitancy across Slovakia.

## 5. Conclusions

Parental hesitancy towards childhood influenza vaccination in Slovakia is a critical public health issue. Our data confirm that this hesitancy is pervasive across all educational backgrounds and is driven primarily by safety concerns and digital misinformation, rather than a lack of general health awareness. Crucially, the pediatrician was identified as the sole robust protective factor against refusal.

From a policy perspective, these findings imply that passive information campaigns are insufficient. We propose that public health strategies must pivot from general education to empowering pediatricians with active communication training. Implementing presumptive communication strategies in primary care could serve as the most effective mechanism to bridge the gap between parental trust and actual vaccination uptake.

## Figures and Tables

**Table 1:** Sociodemographic Characteristics of Survey Respondents and Their Children.

Characteristic	Category	Number (n)	Percentage (%)
**Parent Characteristics (n = 301)**			
Gender	Female	268	89.0
	Male	33	11.0
Age Group (years)	<25	7	2.3
	25–34	139	46.2
	35–44	143	47.5
	45+	12	4.0
Education	University	195	64.8
	Secondary with maturity exam	94	31.2
	Other	12	4.0
**Child Characteristics (N = 443)**			
Age Group (years)	0–2	78	17.6
	5–3 months	116	26.2
	9–6 months	152	34.3
	18–10 months	97	21.9
Number of Children per Family	1 child	141	46.8
	2 children	129	42.9
	3+ children	31	10.3
Families with Preschool-Aged Children (<6 years)	Yes	165	54.8
	No	136	45.2

Note: No statistically significant association was found between parental education level and vaccination intent (*p* > 0.05).

**Table 2:** Key Determinants of Parental Decisions on Childhood Influenza Vaccination by Attitude Group.

Characteristic/Question	Pro-Vaccination Parents (n = 83) [%]	Anti-Vaccination Parents (n = 128) [%]	Hesitant Parents (n = 90) [%]
**Primary Reason for Stance**			
- Protection of child’s health	85.5	N/A	N/A
- Fear of side effects	N/A	57.0	N/A
- Doubts about efficacy	N/A	45.3	N/A
- Need more info on safety/efficacy	N/A	N/A	62.2
- Need clear pediatrician recommendation	N/A	N/A	43.3
**Perception of Influenza Severity**			
- Potentially serious illness	91.6	57.8	68.9
- Common illness	8.4	42.2	31.1
**Perception of Vaccine Safety**			
- Safe	62.7	3.9	11.1
- Have concerns/Not safe	14.5	71.1	35.6
**Perception of Vaccine Efficacy**			
- Sufficiently effective	42.2	0.8	6.7
- Doubts/Low efficacy	3.6	43.0	20.0
**Influence of Pediatrician’s Recommendation**			
- Yes, very much	69.9	35.2	52.2
- Partially	25.3	36.7	43.3
- No	4.8	28.1	4.4

Note: Percentages for reasons for/against and factors for hesitant parents are based on multiple-choice questions and may not sum to 100. N/A indicates the question was not applicable to that group.

**Table 3:** Primary Motivators, Perceived Benefits, and Barriers for Childhood Influenza Vaccination (All Respondents, n = 301).

Category	Specific Factor	Number of Respondents (n)	Percentage (%)
**Top Motivators for Vaccination**			
	Recommendation from a doctor	177	58.8
	Reducing the risk of complications	149	49.5
	Increased information/awareness	117	38.9
	Protection of the child and surroundings	99	32.9
**Top Perceived Benefits of Vaccination**			
	Mitigate the course of influenza	217	72.1
	Prevent influenza illness	169	56.1
	Reduce the risk of complications	141	46.8
	Protect self and surroundings	89	29.6
**Top Concerns/Barriers to Vaccination**			
	Fear of adverse effects	212	70.4
	Doubts about vaccine efficacy	91	30.2
	Lack of information	45	15.0

Note: Percentages are based on multiple-choice questions and may not sum to 100.

**Table 4:** Multivariable logistic regression analysis of determinants of parental willingness to vaccinate.

Predictor	Adjusted Odds Ratio (aOR)	95% Confidence Interval	*p*-Value	Significance
University Education	0.96	0.49–1.88	0.910	ns
Child with Chronic Disease	01.05	0.47–2.31	0.898	ns
Source: Pediatrician	6.67	3.02–14.74	<0.001	***
Source: Internet/social media	0.30	0.15–0.58	<0.001	***
History of Influenza Vaccination	18.84	4.93–72.08	<0.001	***

(ns = not significant; *** = *p* < 0.001. Model adjusted for education, chronic disease status, information sources, and vaccination history. N = 301).

**Table 5:** Multinomial Logistic Regression: Factors distinguishing Hesitant and Accepting parents from Refusers (Reference Category).

Factor	Hesitant vs. Refusers RRR (95% CI)	*p*-Value	Acceptors vs. Refusers RRR (95% CI)	*p*-Value
University Education	1.13 (0.52–2.45)	0.751	1.05 (0.47–2.38)	0.903
Child w/Chronic Disease	0.94 (0.37–2.37)	0.892	1.05 (0.40–2.76)	0.916
Source: Pediatrician	2.45 (1.19–5.04)	0.015	10.99 (4.27–28.28)	<0.001
Source: Internet	0.58 (0.32–1.04)	0.069	0.23 (0.10–0.52)	<0.001
History of Vaccination	1.69 (0.28–10.32)	0.570	21.05 (5.14–86.13)	<0.001

(Reference category = Refusers. RRR = Relative Risk Ratio. Model adjusted for all variables simultaneously. N = 301).

## Data Availability

The raw datasets generated for this study are available on request to the corresponding author. The data are not publicly available due to privacy and ethical restrictions. The full text of the questionnaire administered in this study is included as Supplementary Materials.

## Supplementary Materials

The following supporting information can be downloaded at: https://www.mdpi.com/article/10.3390/children13010144/s1. File S1: Original questionnaire administered to parents (Slovak version); File S2: English translation of the questionnaire.
