# Supplementary material for: Parental Attitudes and Hesitancy Towards Childhood Influenza Vaccination in Slovakia: A Cross-Sectional Survey of 301 Parents
Source: Children (Basel). 2026 Jan 20;13(1):144. doi: 10.3390/children13010144 (PMC12840247; doi:10.3390/children13010144)
Supplement: Supplementary file 1 [file children-13-00144-s001.zip › Supplementary_data_questionnaire_ENG_version.pdf]

**Questionnaire for parents about influenza vaccination**

Thank you for taking the time to complete this questionnaire. Your answers will help us to better understand parents' views on influenza vaccination and to improve awareness of this important topic. Your opinion is valuable and important to us.

This questionnaire is anonymous, so please feel free to answer honestly. Your responses will be processed and used exclusively for research purposes.

** Indicates a required question*

**1. Age of the parent (completing the questionnaire) ***

**2. Gender of the parent (completing the questionnaire) ***

- [ ] Male
- [ ] Female
- [ ] Prefer not to say

**3. Highest educational attainment of the parent (completing the questionnaire):**

- [ ] Primary
- [ ] Secondary
- [ ] University

**4. Child's age in years ***

**5. Number of the child's siblings ***

- [ ] 0
- [ ] 1
- [ ] 2
- [ ] 3
- [ ] 4 or more

**6. Number of siblings of early school age (6 - 11 years) ***

- [ ] 0
- [ ] 1
- [ ] 2
- [ ] 3 or more

**7. Your place of residence (district) ***

**Awareness of influenza and vaccination**

**8. What are your main sources of information about influenza and vaccination? (You can**

**select multiple options)**

- [ ] Doctor
- [ ] Internet
- [ ] Mass media (television, magazines, etc.)
- [ ] Social media (Facebook, Instagram, etc.)
- [ ] Family/friends
- [ ] Other: ________________

**9. As a parent, have you ever been informed about the possibility of influenza vaccination during a visit to the pediatrician? (e.g., during preventive check-ups) ***

- [ ] Yes
- [ ] No
- [ ] I don't remember

**10. Are you aware of the existence of an influenza vaccine (intended exclusively for children aged 2 - 18) with painless administration (nasal spray)? ***

- [ ] Yes
- [ ] No
- [ ] I don't remember

**11. Are you aware that children can be vaccinated against influenza from the age of 6 months? ***

- [ ] Yes
- [ ] No

**12. How well are you informed about influenza in children and its possible complications? (e.g., ear infections, pneumonia, bacterial superinfection) ***

- [ ] Very well
- [ ] Well
- [ ] Average
- [ ] Poorly
- [ ] Not at all

**13. Which groups of people do you think are most at risk of influenza complications? (You can select multiple options) ***

- [ ] Seniors (over 65 years)
- [ ] Chronically ill people (diabetes, cardiovascular diseases, lung diseases)
- [ ] Pregnant women
- [ ] Young children

- [ ] Other: ________________

**14. How well are you informed about influenza vaccination in children and its effectiveness? ***

- [ ] Very well
- [ ] Well
- [ ] Average
- [ ] Poorly
- [ ] Not at all

**15. Do you believe that the influenza vaccination is safe for your child? ***

- [ ] Yes
- [ ] Probably yes
- [ ] I don't know
- [ ] Probably not
- [ ] No

**16. Do you believe that the influenza vaccination is (or could be) effective for your child? ***

- [ ] Yes
- [ ] Probably yes
- [ ] I don't know
- [ ] Probably not
- [ ] No

**17. What are your concerns regarding the influenza vaccination? (You can select multiple options)**

- [ ] Side effects
- [ ] Effectiveness
- [ ] Lack of information
- [ ] Other: ________________

**Experience with influenza vaccination in children**

**18. Has your child been vaccinated against influenza in the past? ***

- [ ] Yes
- [ ] No

**19. If yes, when was your child last vaccinated against influenza? (year)**

**20. If no, why has your child not been vaccinated against influenza? (You can select multiple options)**

- [ ] Concerns about side effects
- [ ] Lack of interest
- [ ] Lack of information
- [ ] Other: ________________

**21. Do you plan to have your child vaccinated against influenza in the future? ***

- [ ] Yes
- [ ] Probably yes
- [ ] I don't know
- [ ] Probably not
- [ ] No

**22. What factors could motivate you to vaccinate your child against influenza? (You can select multiple options) ***

- [ ] Doctor's recommendation
- [ ] Increased awareness about influenza and vaccination
- [ ] Reducing the risk of complications
- [ ] Protecting the child and others
- [ ] Other: ________________

**23. What do you think are the most important reasons for influenza vaccination? (You can select multiple options)**

- [ ] To prevent getting sick with influenza
- [ ] To lessen the course of influenza
- [ ] To reduce the risk of complications
- [ ] To protect oneself and others

**Parents' awareness of influenza**

**24. What do you think are the most common symptoms of influenza? (You can select multiple options)**

- [ ] Fever
- [ ] Cough
- [ ] Sore throat
- [ ] Runny nose
- [ ] Muscle aches
- [ ] Fatigue
- [ ] Other: ________________

**25. Do you think you can distinguish the symptoms of influenza from the symptoms of a common cold? ***

- [ ] Yes
- [ ] No
- [ ] I don't know

**26. What preventive measures against influenza do you usually take? (You can select multiple options)**

- [ ] Frequent hand washing
- [ ] Avoiding contact with sick people
- [ ] Sufficient intake of vitamins (fruits, vegetables)
- [ ] Regular outdoor exercise
- [ ] Cold exposure therapy / Hardening
- [ ] Taking dietary supplements (vitamin C, vitamin D, zinc, probiotics)
- [ ] Other: ________________

**27. Has your child experienced an adverse reaction to the influenza vaccination? ***

- [ ] Yes
- [ ] No

**28. If yes, what was the reaction?**

**29. Has the COVID-19 pandemic influenced your attitude towards influenza vaccination? ***

- [ ] Yes, positively
- [ ] Yes, negatively
- [ ] No, my attitude has not changed
