# Supplementary material for: Parental Attitudes and Hesitancy Towards Childhood Influenza Vaccination in Slovakia: A Cross-Sectional Survey of 301 Parents
Source: Children (Basel). 2026 Jan 20;13(1):144. doi: 10.3390/children13010144 (PMC12840247; doi:10.3390/children13010144)
Supplement: Supplementary file 1 [file children-13-00144-s001.zip › Supplementary_data_questionnaire_SK_version.pdf]

Dotazník pre rodičov o vakcinácii proti chrípke

Ďakujeme, že ste si našli čas na vyplnenie tohto dotazníka. Vaše odpovede nám pomôžu lepšie porozumieť názorom rodičov na **očkovanie proti chrípke** a zlepšiť informovanosť o tejto dôležitej téme. Váš názor je pre nás cenný a dôležitý.

Tento dotazník je **anonymný**, takže sa nemusíte báť odpovedať úprimne. Vaše odpovede budú spracované a použité výlučne pre účely výskumu.

* Označuje povinnú otázku

1. Vek rodiča dieťaťa (ktorý vypĺňa dotazník) *

____

2. Pohlavie rodiča dieťaťa (ktorý vypĺňa dotazník) *

Začiarknite všetky vyhovujúce možnosti.

- [ ] Muž
- [ ] Žena
- [ ] nechcem uviesť

3. Najvyššie dosiahnuté vzdelanie rodiča: (ktorý vypĺňa dotazník) *

Začiarknite všetky vyhovujúce možnosti.

- [ ] Základné
- [ ] Stredoškolské
- [ ] Vysokoškolské

4. Vek dieťaťa v rokoch *

____

5. Počet súrodencov dieťaťa *

Začiarknite všetky vyhovujúce možnosti.

- [ ] 0
- [ ] 1
- [ ] 2
- [ ] 3
- [ ] 4 a viac

6. Počet súrodencov v mladšom školskom veku (6 - 11 rokov) *

Začiarknite všetky vyhovujúce možnosti.

- [ ] 0
- [ ] 1
- [ ] 2
- [ ] 3 a viac

7. Vyše bydlisko (okres) *

____

Informovanosť o chrípke a očkovaní

8. Aké sú vaše hlavné **zdroje informácií** o chrípke a očkovaní? (Môžete označiť viacero možností) *

Začiarknite všetky vyhovujúce možnosti.

- [ ] Lekár
- [ ] Internet
- [ ] Masmédiá (televízia, časopisy a pod.)
- [ ] Sociálne médiá (Facebook, Instagram a pod.)
- [ ] Rodina/priatelia
- [ ] Iné: _______________________________

9. Boli ste niekedy ako rodič dieťaťa **informovaný o možnosti očkovania** proti chrípke počas návštevy u **pediatra** ? (preventívne prehliadky a pod). *

Označte iba jednu elipsu.

- Áno
- Nie
- Nespomínam si

10. Máte informácie o existencii **vakcíny** proti chrípke (určená výlučne pre deti od 2 - 18 rokov) **s bezbolestným podaním** ? (sprej do nosa) *

Označte iba jednu elipsu.

- Áno
- Nie
- Nespomínam si

11. Máte informácie o tom, že očkovať proti chrípke možno už deti od **6 mesiacov** ? *

Označte iba jednu elipsu.

- Áno
- Nie

12. Ako veľmi ste informovaní o chrípke u detí a jej **možných komplikáciách?** (zápal uší, zápal pľúc, bakteriálna superinfekcia a pod.) *

Označte iba jednu elipsu.

- Veľmi dobre
- Dobre
- Priemerne
- Málo
- Vôbec nie

13. Ktoré skupiny ľudí sú podľa vás **najviac ohrozené komplikáciami chrípky**? (Môžete označiť viacero možností) *

Začiarknite všetky vyhovujúce možnosti.

- [ ] Seniori (nad 65 rokov)
- [ ] Chronicky chorí ľudia (diabetes, ochorenia srdca a ciev, pľúcne ochorenia)
- [ ] Tehotné ženy
- [ ] Malé deti
- [ ] Iné: ____________________

14. Ako veľmi ste informovaní o očkovaní proti chrípke u detí a jeho **účinnosti**? *

Označte iba jednu elipsu.

- ( ) Veľmi dobre
- ( ) Dobre
- ( ) Priemerne
- ( ) Málo
- ( ) Vôbec nie

15. Veríte, že očkovanie proti chrípke je pre Vaše dieťa **bezpečné**? *

Označte iba jednu elipsu.

- ( ) Áno
- ( ) Skôr áno
- ( ) Neviem
- ( ) Skôr nie
- ( ) Nie

16. Veríte, že očkovanie proti chrípke je (mohlo by byť) pre Vaše dieťa **účinné**? *

Označte iba jednu elipsu.

- Áno
- Skôr áno
- Neviem
- Skôr nie
- Nie

17. Aké sú vaše **obavy** týkajúce sa očkovania proti chrípke? (Môžete označiť viacero možností) *

Začiarknite všetky vyhovujúce možnosti.

- Nežiaduce účinky
- Účinnosť
- Nedostatok informácií
- Iné: ____________________

Skúsenosti s očkovaním proti chrípke u detí

18. Bolo Vaše dieťa **v minulosti** očkované proti chrípke? *

Označte iba jednu elipsu.

- Áno
- Nie

19. Ak áno, **kedy** bolo vaše dieťa naposledy očkované proti chrípke? (rok)

____

20. Ak nie, prečo vaše dieťa **nebolo očkované** proti chrípke? (Môžete označiť viacero možností)

Začiarknite všetky vyhovujúce možnosti.

- [ ] Obavy z nežiaducich účinkov
- [ ] Nezáujem
- [ ] Nedostatok informácií
- [ ] Iné: ______________________________

21. Plánujete dať **v budúcnosti** vaše dieťa zaočkovať proti chrípke? *

Označte iba jednu elipsu.

- ( ) Áno
- ( ) Skôr áno
- ( ) Neviem
- ( ) Skôr nie
- ( ) Nie

22. Aké faktory by vás mohli **motivovať k očkovaniu** vášho dieťaťa proti chrípke? (Môžete označiť viacero možností) *

Začiarknite všetky vyhovujúce možnosti.

- [ ] Odporúčanie lekára
- [ ] Zvýšená informovanosť o chrípke a očkovaní
- [ ] Zníženie rizika komplikácií
- [ ] Ochrana dieťaťa a okolia
- [ ] Iné: ______________________________

23. Aké sú podľa vás **najdôležitejšie dôvody pre očkovanie** proti chrípke? (Môžete označiť viacero možností) *

Začiarknite všetky vyhovujúce možnosti.

- [ ] Predísť ochoreniu na chrípku
- [ ] Zmierniť priebeh chrípky
- [ ] Znížiť riziko komplikácií
- [ ] Chrániť seba a svoje okolie

Informovanosť rodičov o chrípke

24. Aké sú podľa vás **najčastejšie príznaky chrípky**? (Môžete označiť viacero možností) *

Začiarknite všetky vyhovujúce možnosti.

- [ ] Horúčka
- [ ] Kašeľ
- [ ] Bolesť hrdla
- [ ] Nádcha
- [ ] Bolesti svalov
- [ ] Únava
- [ ] Iné: ____________________

25. Myslíte si, že viete rozlíšiť **príznaky chrípky od príznakov prechladnutia**? *

Označte iba jednu elipsu.

- ( ) Áno
- ( ) Nie
- ( ) Neviem

26. Aké **preventívne opatrenia proti chrípke** bežne vykonávate? (Môžete označiť viacero možností *

Začiarknite všetky vyhovujúce možnosti.

- [ ] Časté umývanie rúk
- [ ] Vyhýbanie sa kontaktu s chorými ľuďmi
- [ ] Dostatočný príjem vitamínov (ovocie, zelenina)
- [ ] Pravidelný pohyb na čerstvom vzduchu
- [ ] Otužovanie
- [ ] Užívanie výživových doplnkov (vitamín C, vitamín D, zinok, probiotiká)
- [ ] Iné: ______________________________

27. Stretli ste sa u vášho dieťaťa s **nežiaducou reakciou** na očkovanie proti chrípke? *

Označte iba jednu elipsu.

- ( ) Áno
- ( ) Nie

28. Ak áno, aká to bola reakcia?

____

29. Ovplyvnila **pandémia COVID-19** váš postoj k očkovaniu proti chrípke? *

Označte iba jednu elipsu.

- ( ) Áno, pozitívne
- ( ) Áno, negatívne
- ( ) Nie, môj postoj sa nezmenil

Tento obsah nie je vytvorený ani schválený spoločnosťou Google.

Google Formuláre
